# Editorial: Computational argumentation: a foundation for human-centric AI

**DOI:** 10.3389/frai.2024.1382426

**Published:** 2024-03-18

**Authors:** Emmanuelle Dietz, Antonis Kakas, Loizos Michael

**Affiliations:** ^1^Airbus Central R&T, Hamburg, Germany; ^2^University of Cyprus, Nicosia, Cyprus; ^3^Open University of Cyprus & CYENS Center of Excellence, Nicosia, Cyprus

**Keywords:** argumentation, human-centric approach, artificial intelligence, formal foundations, learning, reasoning, cognition

## 1 Introduction

What is an appropriate foundation for building Human-centric AI (HCAI) systems? What foundation would allow AI to draw elements from several disciplines to synthesize coherent solutions to the many challenges posed by HCAI?

This research topic stipulates that a foundation for HCAI needs to be at the level of a new underlying logical (reasoning) framework, in an analogous way that Classical Logic is the foundation or Calculus for Computer Science. Resting on the thesis that such a logical framework should be built on a solid understanding of human **cognitive reasoning**, and acknowledging the natural link of argumentation with human cognitive reasoning and human decision making at large, the present research topic explores the proposal of **Argumentation** as the foundation or Calculus for Human-Centric AI (Dietz et al.).

## 2 Call for papers: aim and scope

The aim of this call and its suggestion for the foundational role of argumentation in Human-Centric AI was to help bring together the wide variety of work on argumentation—ranging from argumentation in Philosophy and Ethics to the pragmatics of argumentative discourse in human debates—to understand how to synthesize a viable and robust basis for the development and use of HCAI systems. Systems that would meet their cognitive and ethical requirements, and integrate symbiotically, as expert or peer companions, within the human society, by complementing and enhancing the natural intelligence of humans.

## 3 Research Topic contributions

In addition to the paper that sets the scene for this Research Topic (Dietz et al.), another eight papers were accepted, ranging from results in theoretical work, presentation of own frameworks and setting the context of their work in relation to human-machine interaction in general or with respect to expert domains. Several of the papers have developed own empirical studies serving as an evaluation metric for their frameworks (Albini et al., Kilic et al., Straßer and Michajlova).

Two distinct research directions can be identified among the contributing papers: a direction focusing on theoretical frameworks and development of own empirical studies (Albini et al., Bringsjord et al., Cramer and van der Torre, Straßer and Michajlova), and a direction focusing on the aspects of human-machine interaction and applications to expert domains (such as the medical domain or law) (Bikakis et al., Castagna et al., Kilic et al., Rotolo and Sartor). Yet, all contributions have in common that they agree on the importance of argumentation as foundations for human-centric AI.

### 3.1 Theoretical frameworks and development of own empirical studies

Albini et al. discuss properties of explanations in the context of descriptive accuracy. This implies that explanation contents need to be in correspondence with the internal working of the explained system. The authors provide a formal definitions of naive, structural and dialectical descriptive accuracy using the family of probabilistic classifiers as the context of their analysis. They evaluate their notions by several explanations methods and conduct studies with a varied selection of concrete probabilistic classifiers. Finally, the authors demonstrate how descriptive accuracy could be a critical component in achieving trustworthy and fair systems.

Bringsjord et al. present a new cognitive calculus, in which the central aspect concerns arguments that compete non-monotonically through time. Their framework captures well the three use-case studies, the Monty Hall problem, PERI.2 and the cognitive architecture ARCADIA. Finally, the authors specify seven desiderata for their framework.

Cramer and van der Torre introduce the naive-based argumentation semantics SCF2 and prove that it satisfies two new principles, which are not simultaneously satisfied by any argumentation semantics in the literature. Motivated by findings from empirical studies, these principles seem to correspond well to what humans consider a rational judgment on the acceptability of arguments.

Straßer and Michajlova present a framework for reasoning with higher-order uncertainty. This system integrates with deductive argumentation and can be adjusted to perform well under the so-called rationality postulates of formal argumentation. The authors provide several notions of argument strength, studied both meta-theoretically and empirically by discussing an own empirical study on evaluating argument strength in the context of higher-order uncertainty.

### 3.2 Human-machine interaction and application to expert domains

Bikakis et al. present a visionary paper on the problem of opinion overload in which they argue that it is possibly solvable by exploiting the structure of realistic arguments and understanding an arguer's intentions. The authors identify the main challenges and technological directions, ranging from understanding and formalizing realistic arguments and debates, and developing appropriate models and methods to augmenting Web technologies with the ability to automatically process online arguments. They propose that the realization of this vision will revolutionize Web experience.

Castagna et al. develop EQR (Explanation-Question-Response) argument schemes to generate explanations for treatment advice given to patients in the medical domain using the chatbot, EQRbot. No machine learning algorithm is used, but EQRbot depends on a dynamic knowledge base which is constantly updated with the patient's data.

Kilic et al. focus on expectations and perceptions regarding the role of interaction behavior of a digital companion (with experts and non-experts) in the health domain. They present an empirical requirement elicitation study for an argumentation-based digital companion to support behavior change. The results show that the extent to which a digital companion challenges or supports a user's attitude argumentatively (based on argumentation schemes) can influence the user acceptance and the interaction itself.

Rotolo and Sartor show how explainable AI and legal theory can be modeled in an argumentation framework with structured arguments. The authors review literature of formal models of legal argumentation and investigate the formal connection between argumentation and explanation in law. Their core contribution is the clarification of the structure in normative reasoning of the concepts of justification and explanation through formal argumentation. They argue that the distinction between justification and explanation is pragmatical rather than structural.

## Author contributions

ED: Writing—original draft, Writing—review & editing. AK: Writing—original draft, Writing—review & editing. LM: Writing—original draft, Writing—review & editing.

